# Black Men’s Patient–Clinician Experiences: Pathways to Enhanced Healthcare Outcomes in the United States

**DOI:** 10.3390/healthcare13111230

**Published:** 2025-05-23

**Authors:** Brittany C. Slatton, Kamesha Spates, Maco L. Faniel

**Affiliations:** 1 Department of Sociology, Texas Southern University, Houston, TX 77004, USA; 2 Department of Africana Studies, University of Pittsburgh, Pittsburgh, PA 15260, USA; kspates11@pitt.edu; 3 Independent Researcher, Richmond, VA 23222, USA

**Keywords:** patient–clinician relationships, healthcare experiences, Black men, health equity

## Abstract

**Background/Objectives**: The persistent health disparities affecting Black men in the US healthcare system reflect systemic inequities that impact their health outcomes. This qualitative study employs thematic analysis to examine how Black men’s interactions with medical providers shape their healthcare experiences and to identify key factors influencing their quality of care. **Methods**: Through in-depth interviews with 25 Black men throughout the United States, our thematic analysis identified patterns in their reported healthcare experiences. **Results**: Our analysis revealed four main themes: (1) inadequate clinician communication and information, (2) clinician dismissiveness and failure to listen, (3) experiences of interpersonal racial bias in healthcare interactions, and (4) facilitators of positive, patient-centered healthcare encounters. Black men’s narratives illuminate how communication barriers, dismissive treatment, and racial bias manifest in healthcare settings, while also highlighting elements that facilitate successful patient–clinician relationships. **Conclusions**: The findings suggest specific approaches for improving these interactions, including clinician active listening and bias training, anti-racism medical education, accountability policies, increased clinician diversity, and patient self-advocacy strategies to address systemic factors affecting Black men’s healthcare experiences and outcomes.

## 1. Introduction

Significant health disparities persist for Black men in the United States despite decades of research. A recent longitudinal study has documented alarming statistics, with the Black population experiencing more than 1.63 million excess deaths and over 80 million excess years of life lost compared to the White population from 1999 to 2020 [1]. These disparities are particularly concerning for Black men, who face lower average life expectancy and higher rates of chronic conditions compared to White men [2]. The disparate health outcomes for Black men are well documented. For instance, 42% of Black men have hypertension compared to 31% of White men [3], and they have higher risks for kidney disease and diabetes complications [2,4].

These disparities affect Black men’s physical health as well as the biological aging processes. Research examining biological aging has found evidence that Black Americans experience accelerated “weathering”, a concept developed by Geronimus and colleagues [5] to describe premature aging and health deterioration resulting from chronic exposure to social and economic adversity and political marginalization. A 2018 study finds Black Americans’ biological age averages 2.6 years older than their chronological age, while White Americans’ biological age averages 3.5 years younger than their chronological age, representing a 6.1-year weathering differential between the groups [6]. This weathering effect is amplified by lower socioeconomic status and increased depressive symptoms, which have stronger associations with accelerated aging among Blacks than Whites [6].

Health disparities cannot be attributed to healthcare utilization alone. Recent evidence contradicts stereotypes about Black men’s failure to use healthcare services, showing that they are active users of health services, and they strive to remain healthy for themselves and their families [7]. Additionally, research has found that Black men were more likely to participate in various preventive screenings, including physical examinations, dental check-ups, eye examinations, blood pressure and cholesterol screenings, and cancer screenings, than White men [8]. This evidence challenges stereotypical assumptions about healthcare avoidance among Black men and suggests that barriers to optimal health outcomes extend beyond the issue of healthcare engagement.

Furthermore, Black men’s health disparities persist across socioeconomic strata. Even at equivalent levels of income or education, Black Americans consistently experience worse health outcomes than White Americans [9]. Research indicates that while higher education typically protects Black men from depressive symptoms, Black men who have only completed high school may have a higher risk of depression over time [10]. These findings suggest that social factors beyond traditional socioeconomic variables significantly influence Black men’s health outcomes.

The Institute of Medicine, now known as the National Academy of Medicine (NAM) [11], has acknowledged that even after accounting for socioeconomic differences, race and ethnicity remain significant predictors of disparities in healthcare. The pervasive nature of these disparities points to underlying systemic factors, such as systemic racism, which is a foundational and pervasive system of oppression embedded in all major societal institutions. Systemic racism extends beyond individual prejudice, encompassing centuries-old patterns that subordinate people of color, causing unfair disadvantages [12], such as structured inequalities in resources, power, and opportunity [13]. Systemic racism operates at both social and structural levels, affecting how Black men are perceived and treated within healthcare settings and limiting their access to healthy food, safe neighborhoods, educational and professional opportunities, and quality healthcare [2]. Racism is now widely recognized as a fundamental cause of adverse health outcomes for Black Americans [14].

Medical mistreatment—rooted in systemic racism—has created a deep-rooted mistrust of the healthcare system among many Black men in the United States. This mistrust stems from a history of reproductive exploitation during slavery, the eugenics movements, stereotypes and pseudoscience about Black men’s bodies and sexuality, and a host of medical experimentations—notably the Tuskegee Syphilis Study [15,16,17]. This historical foundation of mistrust continues to influence contemporary healthcare interactions, with research consistently showing that Black patients report lower trust in their physicians than White patients [18,19,20,21,22].

Black men’s lack of trust in healthcare relationships is exacerbated by problematic patient–clinician communication patterns. During clinical encounters with Black patients, physicians have been found to use fewer statements that build rapport and connection, conduct shorter visits, speak more rapidly, and display greater verbal dominance compared to their interactions with White patients [18]. These communication disparities correlate with Black men’s reduced trust and may potentially contribute to the lower rates of healthcare utilization observed among some Black men [11,23,24,25,26,27,28].

Evidence of bias within healthcare settings extends beyond interpersonal interactions to documentation practices. Recent research has found that Black patients had 2.54 times the odds of being described with negative descriptors in their electronic health records compared to White patients, even after adjusting for sociodemographic and health characteristics [29]. These disparities raise concerns about racial bias and the potential transmission of stigma through medical records.

Recent research suggests that media stereotypes often shape healthcare professionals’ perceptions of Black men as “violent, scary, and unreliable”, and these assumptions can affect diagnosis and treatment decisions, including withholding certain procedures or assuming medical non-compliance [30]. In racially discordant medical interactions, non-Black physicians with higher levels of implicit racial bias have been observed using more words reflecting social dominance, such as authoritative statements like “*We need to make sure that you take your medicine*”, and displaying greater anxiety when interacting with Black patients [31].

Implicit biases among healthcare professionals have tangible effects on patient care. A systematic review found that healthcare professionals’ implicit biases were significantly related to patient–clinician interactions, treatment decisions, treatment adherence, and patient health outcomes [32]. A 2016 study found a substantial number of medical students and residents continue to hold false beliefs about biological differences between Blacks and Whites—myths that have historically been used to justify racial oppression. These persistent misconceptions predict racial bias in pain perception and treatment recommendation accuracy, demonstrating how historical race-based pseudoscience continues to influence modern medical practice [33].

The health effects of discrimination extend beyond direct interactions with healthcare providers. Research has associated perceived discrimination with negative physiological responses in Black Americans, including elevated blood pressure that persists during sleep (when it should naturally decrease) and poorer sleep quality with reduced deep sleep compared to White Americans [34,35,36]. These findings support the argument that discrimination significantly contributes to racial differences in important indicators of poor health and disease [37].

Despite ample evidence of systemic bias in the US healthcare system, resistance to addressing it remains. Recent studies with medical students find widespread denial that personal bias can influence clinical outcomes [38]. This resistance highlights the challenges in implementing effective interventions to address healthcare disparities.

The literature clearly establishes that Black men face significant health disparities influenced by systemic factors, historical and ongoing discrimination, and problematic patterns of healthcare interaction. However, relatively few studies have explored Black men’s lived experiences of healthcare encounters from their own perspectives. While survey data from the Pew Research Center indicates that many Black adults report at least one negative interaction with healthcare providers [39], there remains a need for more in-depth qualitative explorations of how Black men navigate and experience healthcare relationships.

The current study addresses this gap by examining Black men’s healthcare experiences through in-depth interviews with participants in the United States. This research investigates the critical question: How do Black men experience and navigate patient–clinician interactions, and what factors contribute to both negative and positive healthcare encounters? By centering Black men’s voices and experiences, this research provides important insights into the challenges they face in healthcare settings and identifies potential pathways for improving care quality and health outcomes. Understanding these experiences is essential not only for improving individual healthcare encounters but also for addressing the persistent health disparities affecting Black men in the United States. By illuminating both the challenges and facilitators of positive healthcare experiences, this research contributes to the development of more effective interventions, policies, and practices to promote health equity for Black men.

## 2. Materials and Methods

### 2.1. Study Design and Participants

Texas Southern University’s Institutional Review Board (IRB) provided approval for this research. To capture the depth and complexity of Black men’s healthcare experiences, we employed a qualitative study design using in-depth interviews. This approach allowed for a detailed exploration of participants’ lived experiences [40]. Eligibility criteria for study participation included self-identification as Black or African American, male, at least 18 years of age, and residence within the United States. The first and third authors used purposive sampling techniques to recruit 25 participants through professional social media channels like LinkedIn and community referral networks. Of approximately 300 Black men who received invitations to participate in the study, about 150 responded to the initial recruitment advertisement; around 75 respondents were subsequently excluded for not meeting inclusion criteria (such as not residing in the United States), while others discontinued participation of their own volition, resulting in our final sample of 25 participants. Participants engaged in the study only after providing written and verbal informed consent, which detailed study objectives, confidentiality protections, and their right to discontinue participation without consequences. Participants received a USD 50 gift card in acknowledgment of their time and insights.

### 2.2. Interview Process

The third author facilitated all in-depth interviews remotely via Zoom between October 2023 and April 2024. Each conversation lasted approximately 40 min and was digitally recorded with participant authorization. The interview protocol included open-ended questions, such as “Do you trust your doctors to make medical decisions that are in your best interest?”, “Have your doctors ever refused to provide you with appointments or treatment?”, “Have your doctors ever refused to provide you with pain medication?”, “Do you believe you have experienced discrimination from doctors, nurses, or other healthcare professionals?”, and “Do you feel that you were ever treated differently by healthcare providers specifically because you are a Black man?” Participants were asked to explain their experiences and probed for additional relevant information.

The third author, race and gender concordant with the participants, conducted the interviews using culturally responsive techniques that prioritized rapport-building and created safe spaces for authentic sharing. Recognizing historical mistrust toward research among Black men, he approached interviews as collaborative exchanges rather than extractive processes. This approach included obtaining thorough informed consent, asking thoughtful follow-up questions, and occasionally sharing relevant personal reflections to foster trust and engagement while empowering participants in their narrative sharing.

### 2.3. Data Management

We used the automated transcription service, Transcribe, to convert audio recordings to text. The transcription service has strict access protocols and comprehensive security measures, including restricted access controls and data encryption. All identifying information was removed from transcripts prior to uploading audio recordings to the transcription service. Upon completion of converting the audio to written transcripts, all audio recordings were deleted from the Transcribe system. To ensure confidentiality, the first author maintained all research materials, including audio recordings and transcripts, on a secure, password-protected computer in a locked private office.

### 2.4. Data Analysis

The analytical process followed Braun and Clarke’s six-phase thematic analysis framework [41]. We implemented this methodology through a systematic process beginning with data familiarization through repeated readings of transcripts; developing initial codes inductively from participants’ language rather than predetermined categories; generating preliminary themes by identifying patterns across codes; refining themes through iterative comparison with the dataset; defining and naming themes to capture their essence and relationships; and, finally, producing the analytical report. An independent qualitative methodologist and the first author conducted analyses of the data. Initial coding was performed independently on all transcripts using an open coding approach where codes emerged directly from participants’ experiences rather than predetermined frameworks. The process began with multiple careful readings of each transcript, followed by systematic line-by-line coding to identify meaningful segments related to healthcare experiences. The codes were compared, and minor discrepancies were resolved through discussion to develop a comprehensive coding framework. The qualitative software NVivo (version 14) was used to facilitate the organization and coding of the data. The codes were then organized into potential themes and subthemes based on patterns of meaning. This process included searching for negative cases that contradicted emerging patterns and revising thematic boundaries to accommodate the full range of participant experiences. The second and third authors then evaluated these preliminary themes for consistency with participant narratives, theoretical relevance, and internal coherence. Final themes emerged through iterative refinement until reaching theoretical saturation, which is the point at which additional analysis yielded no new thematic elements.

## 3. Results

### 3.1. Participant Characteristics

This qualitative study included 25 Black male participants with diverse demographic characteristics. The sample consisted of adults primarily aged 30–39 (41.2%) and 18–29 (35.3%), with the majority (82.4%) living in the South. Most participants were highly educated, with 47.1% holding master’s degrees and 11.8% with doctorate degrees. In terms of income, the largest proportion of participants (47.1%) earned between USD 60,000 and USD 99,999 annually. Most participants were employed, with 58.8% working full-time (40 or more hours weekly) and 35.3% working part-time. It should be noted that approximately one-third of participants had missing demographic data; these cases were excluded from percentage calculations to provide a more accurate representation of the reported demographics (Table 1).

### 3.2. Themes and Representative Quotes

Through thematic analysis of in-depth interview data, we identified four predominant themes reflecting participants’ healthcare experiences with medical providers: (1) inadequate clinician communication and information—participants described receiving insufficient explanations about medical conditions and treatment options; (2) clinician dismissiveness and failure to listen—men recounted instances where medical concerns were minimized or disregarded; (3) experiences of interpersonal racial bias in healthcare interactions—participants reported experiencing differential treatment based on racial identity; and (4) facilitators of positive, patient-centered healthcare encounters—men highlighted elements that enhanced their healthcare interactions, including respectful communication and cultural awareness. Table 2 provides illustrative quotes for each theme.

### 3.3. Theme 1. Inadequate Clinician Communication and Information

Participants consistently described experiences where healthcare providers failed to deliver adequate communication or sufficient information during clinical encounters. This theme manifested in several ways, including rushed appointments, limited explanations of conditions or treatments, and failure to follow up. Several men expressed frustration with the brevity of clinical encounters and how this impacted the quality of care they received. One participant with extensive professional experience in healthcare compliance highlighted how the abbreviated appointment directly contradicted standard billing practices:

*“I do have a health care compliance background, certifications, and all that kind of stuff… The level of service that you’re providing or what you’re documenting is not equal to the code that you’re using because I didn’t get 30 min of intensive services. I got less than 15 min”*

(Participant 14)

This participant’s background in healthcare compliance and auditing for a large mental health organization provided him with unique insight into the discrepancy between the level of service charged and what was actually delivered. His observation underscores how shortened appointment times directly impact providers’ ability to deliver comprehensive information and engage in meaningful communication.

Participants also reported concerns about insufficient diagnostic effort and inadequate explanations of medical conditions. One participant expressed: “I would say no in some regards to, um, maybe feeling like doctors aren’t taking additional steps that might be necessary to diagnose whatever my symptoms are” (Participant 20). The perception that providers were not conducting thorough examinations or investigations of symptoms was tied to concerns about communication quality. Another participant elaborated on this connection:

*“I would say not enough communication… sometimes you go to the doctor, they’ll say, okay. I think you have this. Prescribe you some medicine. They may say a few things about what’s going on…And just updating and just, you know, seeing how you’re feeling, um, after you get the medicine, um, just it’s, it’s the communication isn’t as well as I believe it should be”*

(Participant 10)

This same participant emphasized that spending additional time with patients could lead to a more thorough understanding of their conditions: “… But, you know, I feel as if sometimes if a doctor could just spend a little bit more time with the patient and, uh, go further into what’s going on with the patient” (Participant 10). The need for clarification and the suspicion that physicians might be providing rushed responses was another common aspect of this theme. One participant described his ongoing need to verify information received from his doctor:

*“Um, I think there’s still, like, some hesitation with things, um, like, when I communicate with my doctor. Um, I need a lot of clarification or there’s a lot of questioning that comes from, you know, feedback or just making sure that he is being attentive to the things that I bring to him. Um, and not just trying to fill me a quick answer to get me in and get me out”*

(Participant 16)

This participant’s experience reflects his underlying concern that his doctor may not be fully attentive to his concerns and might prioritize efficiency over thoroughness, leading to inadequate communication of important health information. For some participants, inadequate communication during standard appointments led them to seek additional care from providers they felt might be more attentive. One veteran described his experience with a non-Black physician in the VA system:

*“…I go to the appointments and the standard checklist. You know, I communicate to her what I need… Kinda to be focused on, but, you know, like, she asked me questions, and and I feel like there should be more that comes out of it [the appointment]. But it, it doesn’t, and I’m like, okay. Well, you know, I’ll follow-up with my my PCP [who is Black]”*

(Participant 14)

This participant had established a relationship with a Black primary care physician outside the VA system specifically to address the communication gaps he experienced with his VA provider. His experience highlights how inadequate clinician communication can lead patients to seek additional care, potentially fragmenting their healthcare experience and creating inefficiencies in the system.

### 3.4. Theme 2. Clinician Dismissiveness and Failure to Listen

Many participants described encounters where clinicians appeared to dismiss their concerns or failed to listen attentively to their descriptions of symptoms, medical history, or healthcare needs. This theme manifested through providers’ disregard of patient-reported information, inappropriate assumptions, and rushed interactions that left participants feeling unheard and undervalued. Several participants expressed frustration with providers who seemingly disregarded their input about their own health. One participant compared physicians to mechanics using a trial-and-error approach:

*“Oh, I mean, I’ve, I feel like doctors don’t know it all. They’re more like, you know, mechanics where it’s kinda like trial and error… they’re trying to get to the problem, but they don’t… And sometimes they don’t hear you when you tell them what the [problem is]”*

(Participant 9)

This analogy highlights the participant’s perception that physicians often rely on standardized diagnostic approaches rather than carefully considering the patient’s own assessment of their condition. One particularly concerning example of provider failure to listen involved a participant with a family history of prostate issues who repeatedly had his medical concerns misattributed to sexually transmitted infections:

*“I knew I, I wasn’t having sex. I knew I didn’t have an STD, so the test came back negative. Um, went to the clinic again. They said, oh, take another one [STD test]. Came back negative…Went to the urologist, and the urologist was…making jokes, you know, telling me, you know, wrap it up… So he gave me another [STD] test, um, and it came back negative…. I kept telling him, my dad has prostate issues. You know, it runs in my family…. I just was telling him, hey, man. I don’t think I have a STD…And, you know, he just continued to keep making jokes”*

(Participant 10)

This participant’s experience reveals how providers repeatedly ignored his accurate reporting of his sexual history and family medical history. Despite multiple negative STD tests, healthcare providers continued to pursue that diagnostic path while making inappropriate jokes, rather than considering the prostate concerns he had specifically raised based on his family history. This case illustrates how failure to listen can lead to delayed diagnosis, unnecessary testing, and significant patient distress. Participants also reported witnessing family members experiencing provider dismissiveness. The same participant described his father’s frustrating experiences seeking care:

*“…My dad…has gone through cancer screenings, uh, prostate, um, doctor after doctor… it’s the same different medicine, different medicine. Um, one time they told him it was his wallet that was causing muscle spasms”*

(Participant 10)

The trivializing explanation of a wallet causing symptoms that warranted cancer screenings exemplifies how serious health concerns can be minimized by providers, potentially delaying appropriate treatment. Pain dismissal emerged as a particularly troubling form of provider dismissiveness. One participant succinctly described this experience. “Your pain gets dismissed, you know, the conversation kind of, like, it feels like it’s being expedited” (Participant 19). This account connects pain dismissal with rushed clinical encounters, suggesting that providers may prioritize efficiency over thoroughly addressing patients’ discomfort or concerns.

Beyond physical symptoms, participants reported that their broader healthcare needs were sometimes dismissed. A participant who takes pre-exposure prophylaxis (PrEP) for HIV prevention described being discouraged from regular in-person visits with his physician:

*“So, I was very frustrated, um, very hurt, or I felt very dismissed, um, because I was going to my regular 90-day appointment… he told me, um, I would say in September of 2022, hey. Don’t come back. Just do your blood work… I wanted to do an in person visit. [The doctor said] ‘I told you, you didn’t have to come back, just the, you know, get the HIV test. You don’t need to come back.’ So, I stopped going because he, it’s like he didn’t wanna see me”*

(Participant 6)

This participant’s experience reveals how his desire for comprehensive healthcare was reduced to HIV testing alone. The physician’s apparent unwillingness to conduct regular in-person appointments for general health concerns left the participant feeling unwanted and ultimately led him to discontinue care. This example illustrates how provider dismissiveness can directly impact care continuity and potentially lead to unaddressed health issues.

### 3.5. Theme 3. Experiences of Interpersonal Racial Bias in Healthcare Interactions

Participants described numerous experiences that revealed racial bias in their healthcare encounters. These biases manifested through stereotyping, differential treatment, and lowered expectations based on race. The accounts revealed how racial assumptions influenced clinical decision-making and patient–clinician interactions. Participants reported encountering stereotypes about drug use when seeking medical care. A participant articulated this experience: “…there’s an assumption that you don’t know your body. And if something’s wrong with you, it’s because you did drugs. And because you’re a Black person, you you must have did drugs” (Participant 25). This statement highlights the dual burden of having expertise about one’s own body dismissed while simultaneously being subjected to racialized assumptions about substance use. A participant with sickle cell disease described experiencing stigma when seeking pain management:

*“My first reaction, and this may be biased because my mom is in the mental…health field as a nurse, um, is that there is a stigma behind, um, those who have sickle cell and being, uh, narcotic seeking”*

(Participant 5)

His observation reflects the well-documented phenomenon of pain treatment disparities for Black patients, particularly those with conditions like sickle cell disease that require pain management. Despite having a legitimate medical need for pain relief, he encountered suspicion that his requests were motivated by addiction rather than genuine medical necessity.

Participants also described experiences of being devalued within the healthcare system. One participant suggested that providers invest less care when treating patients from certain demographic backgrounds:

*“And I think maybe health care professionals, um, almost, uh, like, subconsciously pay less when treating lower income Black people or people of color because they think that they might be wasting their time”*

(Participant 21)

This perception of receiving lower quality care due to race and socioeconomic status was echoed in experiences of being overlooked in clinical settings. A participant described witnessing apparent racial prioritization in emergency room settings:

*“I’ve…been to, uh, to an emergency room setting to…not bleeding or anything, but with, uh, you know, maybe some, uh, severe pain or something. And I’ve seen them [ER doctors/nurses] actually skip over or attempt to skip over me, uh, clearly when I was there before [in favor of] uh, you know, other ethnicities, White, Mexican”*

(Participant 12)

Beyond treatment decisions, participants reported experiencing judgment based on their physical appearance. One participant noted how his hairstyle prompted assumptions from healthcare providers:

*“…For instance, my hair—dreads—you know…people who are, um, non-Black…even, you know, certain older Black…people who…provide medical service for me will also make comments because they might think that my hairstyle or my dress style…promotes a particular thing about me”*.

(Participant 19)

This account demonstrates how cultural expressions through appearance can trigger biased responses from healthcare providers, potentially affecting the quality of care received. Perhaps most troubling were instances where providers expressed lowered expectations for Black patients’ capabilities and achievements. A participant with sickle cell disease described a pattern of discouragement from healthcare providers throughout his academic journey:

*“… I had asked them [doctors] for a letter of accommodation for my undergraduate [program]…and they were like… ‘that’s great. I can totally do that. Uh, just know that it’s really difficult for people with sickle cell to be able to complete an undergrad.’ Got my undergrad, went into masters, needed the letter for the new university. They’re like: ‘Wow, I’m surprised that you’re able to get a master’s. So, it’s gonna be very difficult for you…don’t be surprised if you don’t make it.’ …. And then, again, PhD, same kind of comments. And while that may be rooted in their research of sickle cell, um, I also believe that it is because I’m a Black male”*

(Participant 5)

While providers presented their pessimistic outlook as being informed by medical knowledge about sickle cell disease, the participant recognized a pattern that suggested racial bias was also influencing their assessment of his capabilities. The consistent expressions of surprise at his academic achievements and predictions of future failure reveal how implicit racial bias may shape provider expectations and potentially limit supportive care.

### 3.6. Theme 4. Facilitators of Positive, Patient-Centered Healthcare Encounters

While participants identified numerous barriers to quality healthcare, they also described factors that facilitated positive healthcare experiences. These positive factors centered around two main subthemes: race concordance with medical providers and provider investment in relationship building. Race concordance with healthcare providers emerged as a significant factor in creating positive healthcare experiences. Many participants described intentionally seeking out Black physicians or physicians of color: “…all of my medical providers right now are Black, with the exception of [one]. So, I’ve been very, like, intentional about that” (Participant 19). This deliberate selection of Black providers was often based on participants’ belief that these providers would have greater cultural understanding and insight into health concerns specific to Black patients. A participant said the following:

*“I do now [have a Black doctor] because my wife and I switched to a, um, a Black, um, primary care physician… And she seems to have more insight, uh, directly as African American, uh, health concerns and things of that nature…And so, I think changing to a Black primary care physician has increased the trust”*

(Participant 2)

The connection between provider race and trust was explicitly mentioned by several participants. Historical awareness of racial disparities in healthcare influenced these preferences. One participant noted “…And, I mean, I generally choose doctors of color that I think understand, um, the experience of people of color in the medical industry” (Participant 21). Another participant referenced the historical mistreatment of African Americans in healthcare as a motivation for seeking providers of color:

*“And then on top of that, um, because I know the experiences of, um, like, African Americans in mistreatment in terms of the medical profession. That’s why, I am very intentional to have a person of color as my doctor”*

(Participant 5)

Beyond race concordance, participants emphasized the importance of provider investment and relationship building in creating positive healthcare experiences. The development of rapport with healthcare providers was identified as critical to satisfaction with care:

*“Um, currently, I would say yes. I actually feel pretty good about the doctors that I have now… I’ve had different experiences over over time… but the I have a specialist for the Crohn’s issues, and I have a general, uh, primary care doctor, both of which I’ve had pretty good experiences with and built a pretty good kinda rapport with”*

(Participant 7)

This same participant elaborated on what distinguished positive provider relationships from negative ones:

*“But anyway, the the difference for me, I think, had a lot to do with the level of, like, connection. So, like, you know, just talking to me and, uh, remembering me. And I think showing a level of investment and, like, exploring different things and trying to figure out how to get to a better place than where I was when I first started going there”*

(Participant 7)

The experience of feeling that a provider was genuinely engaged rather than rushing through an appointment contributed significantly to positive evaluations of care. A participant stated the following:

*“They [doctors] were from the Bronx… I felt more comfortable, and it felt like they were actually trying… it didn’t feel like somebody was trying to quickly finish a session. It felt like they were trying to make sure that I had, um, it’s it’s kinda like how you asked the follow-up question”*

(Participant 19)

The primary factors identified as promoting positive healthcare experiences—race concordance and provider investment in relationship building—directly counteract the negative themes of inadequate communication, dismissiveness, and racial bias described in earlier themes. These findings highlight actionable approaches that could improve healthcare experiences for Black men.

## 4. Discussion

This study explored Black men’s interactions with clinicians, revealing three themes that negatively affected their care experiences: inadequate clinician communication, dismissive attitudes, and interpersonal racial bias. These findings illuminate the complex dynamics undermining healthcare quality for Black men, which can contribute to persistent health disparities. Participants also identified a fourth theme—facilitators of positive, patient-centered healthcare encounters—which provides valuable direction for improving care delivery. Our findings align with and extend the existing literature on healthcare disparities affecting Black men. The inadequate communication and dismissiveness themes parallel research by Martin et al. [18], who found physicians use fewer rapport-building statements with Black patients, resulting in shorter visits and reduced trust. Communication breakdown directly impacts health outcomes through decreased medication adherence [42,43], delayed follow-up care [44], and fragmented healthcare experiences. Similarly, participants’ experiences of dismissiveness, particularly regarding pain and symptoms, connect to documented patterns of provider behavior that contribute to diagnostic delays and inappropriate treatment plans [33,45]. These experiences reflect broader patterns of implicit bias in healthcare, where providers’ unconscious biases influence clinical decision-making [32], creating systemic barriers to equitable care. Hoffman et al. [33] demonstrated how false beliefs about biological differences contribute to racial bias in pain perception and treatment recommendations, directly impacting the pain management experiences described by our participants with conditions like sickle cell disease. Conversely, the positive experiences identified in our study align with research showing that race-concordant care and patient-centered approaches lead to improved health outcomes, such as higher life expectancy [46]. These connections between patient–clinician interactions and health outcomes underscore the critical importance of addressing interpersonal and systemic factors in healthcare delivery for Black men.

Overall, the findings illustrate how interpersonal clinical interactions can reflect and perpetuate broader patterns of structural racism within healthcare delivery systems, contributing to experiences that shape Black men’s access to quality care [13,47]. The communication barriers, dismissive treatment, and racial bias Black men encounter are not random events but consistent patterns that reveal how structural inequities in healthcare directly affect their lived experiences. When healthcare professionals minimize symptoms, rush appointments, or make biased assumptions, they reinforce institutional practices that limit Black men’s access to comprehensive diagnostics, appropriate pain management, and consistent care—directly contributing to documented disparities in chronic disease [2,4], preventive screening utilization [8], and mortality rates [1]. By understanding these patient–clinician interactions as expressions of structural factors rather than individual failings, we can develop more effective interventions that address both the interpersonal dynamics and the underlying systems that perpetuate health inequities for Black men.

Participants highlighted two key factors contributing to positive clinical interactions: race-concordant care and clinicians who demonstrate genuine engagement by investing time, showing respect, and thoroughly addressing patient concerns. Building on the experiences of our participants, we suggest several targeted recommendations across multiple levels of the healthcare system to address the challenges encountered by Black men. These recommendations focus on actionable strategies for clinicians, medical education institutions, policymakers, and patient advocacy programs to collectively improve Black men’s healthcare experiences and outcomes.

Clinicians can enhance patient-centered communication with Black men by prioritizing active listening, which is considered the most effective form of listening [48,49] and entails “avoiding interruption, maintaining interest, postponing evaluation, organizing information, and showing interest” [48]. Active listening could help clinicians understand their Black patients’ needs, fostering better communication and building essential trust. Practical applications include scheduling brief follow-up contacts between visits, creating collaborative care plans that incorporate patient priorities, and including verified treatment barriers reported by patients, even if briefly, in their notes. Within ethical and legal boundaries, clinicians may also consider implementing adherence programs that include non-financial incentives to promote patient treatment and follow-up care. Clinicians should also allocate sufficient time for appointments with Black men, ensuring thorough explanations of diagnoses, treatment options, and follow-up care. As our participants noted, rushed appointments significantly undermined trust and satisfaction with care. Implementing communication techniques that emphasize partnership rather than authority [31] could help establish more equitable relationships. Clinicians could benefit from annual continuing education courses that enhance communication skills with diverse patients. Regular self-assessment through instruments like the Implicit Association Test (IAT) can help clinicians identify and address biases that may influence their clinical decisions [50].

Medical education should integrate anti-racism training throughout curricula, rather than as isolated modules. This training should directly address the false beliefs about biological differences between races [33] and explain how historical misconceptions about race continue to influence modern practice and contribute to disparities in pain management and treatment recommendations. Medical education should incorporate the lived experiences of Black patients through case studies and patient narratives. For example, participants’ accounts of clinician microaggressions—brief verbal or non-verbal disparagements [51,52,53], including dismissiveness, biased assumptions, and inappropriate jokes—should be integrated into medical training. Exposing students to these experiences and their impact on patient trust and care continuity may enhance empathy and foster cultural humility.

Healthcare policy should mandate accountability measures to address racial disparities in care quality. State policymakers should require healthcare institutions to implement standardized bias and discrimination reporting systems with data submitted to a central repository. Such systems could be modeled after the Bias Reporting Tool (BRT) developed by UW Medicine in Seattle, Washington [54]. These systems should include clear evaluation metrics, including analysis of reported incidents and patient satisfaction surveys, to measure progress. Implementing this approach across states would enable the identification of systemic patterns requiring targeted intervention. Policymakers should also establish targeted funding streams and incentive programs to increase racial diversity among clinicians, which would expand patient access to race-concordant care. To address retention and prevent burnout of clinicians of color, policymakers should create financial incentives for healthcare institutions that implement peer support networks, provide dedicated mental health resources, ensure reasonable workload distributions, and create “safe space” groups where professionals of color can share experiences and strategies for navigating workplace challenges. These initiatives should also support the recruitment of Black healthcare professionals through structured mentorship opportunities and protected time for community engagement initiatives. This recommendation is supported by our finding that participants actively sought race-concordant care and is consistent with research demonstrating how racial concordance improves patient trust and adherence to treatment recommendations [55].

Equipping Black men with effective self-advocacy strategies is essential for navigating potentially dismissive or biased healthcare encounters. Healthcare organizations should partner with trusted community centers and churches to develop and distribute culturally relevant self-advocacy resources to Black men and train health workers who can connect clinical care to their everyday experiences. Self-advocacy resources, such as question guides, patient rights information, and approaches for seeking second opinions, can empower Black men to secure thorough examinations, clear explanations, and appropriate care. Recent research underscores the value of these approaches, showing that patient self-advocacy can reduce the impact of physicians’ implicit biases [56], making these tools particularly valuable for Black men seeking equitable healthcare. Patient advocacy organizations should collaborate with healthcare institutions to establish patient advisory boards with significant representation from Black men. These boards could provide input on policies, procedures, and communication practices to ensure they address the concerns highlighted in our study.

### Limitations

While the study offers rich insights into Black men’s healthcare experiences, certain methodological and contextual limitations must be considered. First, the research team focused exclusively on Black men aged 18 and above. This approach may not depict the full spectrum of experiences across socioeconomic status, sexual orientation, disability, or geographical location. Future research should address this shortcoming by adopting an intersectional framework to offer a more comprehensive perspective of within-group experiences of Black men. Next, the research team conducted interviews with twenty-five Black men for this study. Although our focus was on obtaining detailed insights into their communication experiences with healthcare providers, we acknowledge that the limited sample size may restrict the generalizability of our findings beyond this group. Additionally, the recruitment of participants was facilitated through our social media platforms and personal networks. This approach may introduce selection bias, thereby further limiting the external validity of our results. Future research should consider addressing these limitations by conducting large-scale studies that use a national random sample, as well as longitudinal studies that track changes in Black men’s healthcare experiences over time. Lastly, the narratives shared by Black men for this study were reliant on retrospective self-reporting, which may introduce recall bias as participants attempt to recall their past healthcare experiences. Future research may consider studies that employ ecological momentary assessment approaches that collect real-time data through daily mobile phone surveys or health diaries to mitigate recall bias and provide a more comprehensive understanding of their experiences. Additionally, the implementation of scientific studies is recommended to evaluate the effectiveness of the recommendations this study proposes across various healthcare levels.

## 5. Conclusions

This study offers important insights into the healthcare experiences of Black men, highlighting significant challenges related to provider communication, dismissiveness, and racial bias. By centering Black men’s voices and perspectives, we have identified both barriers to quality care and facilitators of positive, patient-centered healthcare experiences. The findings underscore the need for multifaceted interventions addressing individual provider behaviors, medical education, healthcare policy, and patient empowerment. To effectively transform Black men’s healthcare experiences, concrete actions must be implemented at multiple levels. Healthcare systems should mandate bias reporting systems and clinician diversification initiatives, medical education must integrate anti-racism training with case-based learning on microaggressions, practicing clinicians should adopt evidence-based communication protocols that prioritize active listening and partnership, and policymakers must establish accountability frameworks that tie quality measures to equitable care outcomes. The significance of this research lies in its qualitative approach that captures the nuanced ways Black men experience healthcare interactions beyond what health disparities data can reveal. These findings are particularly important given the persistent underrepresentation of Black men’s perspectives in health research and the historical tendency to focus on behavioral rather than structural explanations for disparities. Addressing these disparities requires acknowledging the historical context of medical racism while implementing systemic changes to dismantle persistent barriers to healthcare equity for Black men.

## Figures and Tables

**Table 1:** Respondent demographics.

Characteristics	Respondents (n = 25)	%
Age Group		
18–29	6	35.3
30–39	7	41.2
40–49	4	23.5
Region		
South	14	82.4
Northeast	3	17.6
Education		
High school degree or equivalent	2	11.8
Some college	2	11.8
Bachelor’s degree	3	17.6
Master’s degree	8	47.1
Doctorate degree	2	11.8
Income		
USD 0–29,999	3	17.6
USD 30,000–59,999	3	17.6
USD 60,000–99,999	8	47.1
USD 100,000 or more	3	17.6
Employment		
Employed, working 1–39 h per week	6	35.3
Employed, working 40 or more hours per week	10	55.8
Not employed, looking for work	1	5.9
Missing/No Response (all variables)	8	
Total Sample	25	

**Table 2:** Themes and representative quotes.

Themes	Representative Quotes
1. Inadequate Clinician Communication and Information	“…I do have a health care compliance background, certifications, and all that kind of stuff… The level of service that you’re providing or what you’re documenting is not equal to the code that you’re using because I didn’t get 30 min of intensive services. I got less than 15 min.”
	“I would say no in some regards to, um, maybe feeling like doctors aren’t taking additional steps that might be necessary to diagnose whatever my symptoms are.”
	“I would say not enough communication… sometimes you go to the doctor, they’ll say, okay. I think you have this. Prescribe you some medicine. They may say a few things about what’s going on…And just updating and just, you know, seeing how you’re feeling, um, after you get the medicine, um, just it’s it’s the communication isn’t as well as I believe it should be.”
	“Um, you know, sometimes you go to the doctor, they’ll say, okay. I think you have this… But, you know, I feel as if sometimes if a doctor could just spend a little bit more time with the patient and, uh, go further into what’s going on with the patient.”
	“Um, I think there’s still, like, some hesitation with things, um, like, when I communicate with my doctor. Um, I need a lot of clarification or there’s a lot of questioning that comes from, you know, feedback or just making sure that he is being attentive to the things that I bring to him. Mhmm. Um, and not just trying to fill me a quick answer to get me in and get me out.”
	“…I go to the appointments and the standard checklist. You know, I communicate to her what I need… Kinda to be focused on, but, you know, like, she asked me questions, and and I feel like there should be more that comes out of it [the appointment]. But it it doesn’t, and I’m like, okay. Well, you know, I’ll follow-up with my my PCP [who is Black].”
2. Clinician Dismissiveness and Failure to Listen	“Oh, I mean, I’ve, I feel like doctors don’t know it all. They’re more like, you know, mechanics where it’s kinda like trial and error… they’re trying to get to the problem, but they don’t Yeah. And sometimes they don’t hear you when you tell them what the [problem is].”
	“I knew I I wasn’t having sex. I knew I didn’t have an STD, so the test came back negative. Um, went to the clinic again. They said, oh, take another one [STD test]. Came back negative…Went to the urologist, and the urologist was…making jokes, you know, telling me, you know, wrap it up… So, he gave me another [STD] test, um, and it came back negative…. I kept telling him, my dad has prostate issues. You know, it runs in my family…. I just was telling him, hey, man. I don’t think I have a STD…And, you know, he just continued to keep making jokes.”
	“…My dad…has gone through cancer screenings, uh, prostate, um, doctor after doctor… it’s the same different medicine, different medicine. Um, one time they told them it was his wallet that was causing muscle spasms.”
	“Your pain gets dismissed, you know, the conversation kind of, like, it feels like it’s being expedited.”
	“So, I was very frustrated, um, very hurt, or I felt very dismissed, um, because I was going to my regular 90-day appointment… he told me, um, I would say in September of 2022, hey. Don’t come back. Just do your blood work… I wanted to do an in person visit. [The doctor said] ‘I told you you didn’t have to come back, just the, you know, get the HIV test. You don’t need to come back.’ So, I stopped going because he it’s like he didn’t wanna see me.”
3. Experiencing Interpersonal Racial Bias in Healthcare	“Yeah. They want there’s an assumption that you don’t know your body. And if something’s wrong with you, it’s because you did drugs. And because you’re a Black person, you you must have did drugs.”
	“My first reaction, and this may be biased because my mom is in the mental…health field as a nurse, um, is that there is a stigma behind, um, those who have sickle cell and being, uh, narcotic seeking.”
	“And I think maybe health care professionals, um, almost, uh, like, subconsciously pay less when treating lower income Black people or people of color because they think that they might be wasting their time.”
	“I’ve…been to, uh, to an emergency room setting to, you know, not bleeding or anything, but with, uh, you know, maybe some, uh, severe pain or something. And I’ve seen them (ER doctors/nurses) actually skip over or attempt to skip over me, uh, clearly when I was there before [in favor of] uh, you know, other ethnicities, White, Mexican.”
	“…For instance, my hair—dreads—you know…people who are, um, non-Black…even, you know, certain older black…people who…provide medical service for me will also make comments because they might think that my hairstyle or my dress style…promotes a particular thing about me.”
	“… I had asked them [doctors] for a letter of accommodation from my undergraduate…and they were like… ‘that’s great. I can totally do that. Uh, just know that it’s really difficult for people with sickle cell to be able to complete an undergrad.’ Got my undergrad, went into masters, needed the letter for the new university. They’re like: ‘Wow, I’m surprised that you’re able to get a master’s. So, it’s gonna be very difficult for you…don’t be surprised if you don’t make it.’ …. And then, again, PhD, same kind of comments. And while that may be rooted in their research of sickle cell, um, I also believe that it is because I’m a Black male.”
4. Facilitators of Positive, Patient-Centered Healthcare Encounters	“I have all all of my medical providers right now are Black, with the exception of [one]. So, I’ve been very, like, intentional about that…”
	“I do now because my wife and I switched to a, uh, a Black, um, primary care physician… And she seems to have more insight, uh, directly as African American, uh, health concerns and things of that nature…And so I think changing to a Black primary care physician has increased the trust.”
	“…And, I mean, I generally choose doctors of color that I think understand, um, the experience of people of color in the medical industry.”
	“And then on top of that, um, because I know the experiences of, um, like, African Americans in mistreatment in terms of the medical profession, that’s why, I am very intentional to have a person of color as my doctor.”
	“Um, currently, I would say yes. I actually feel pretty good about the doctors that I have now… I’ve had different experiences over over time… but the I have a specialist for the Crohn’s issues, and I have a general, uh, primary care doctor, both of which I’ve had pretty good experiences with and built a pretty good kinda rapport with.”
	“But anyway, the the difference for me, I think, had a lot to do with the level of, like, connection. So, like, you know, just talking to me and, uh, remembering me. And I think showing a level of investment and, like, exploring different things and trying to figure out how to get to a better place than where I was when I first started going there.”
	“They were from the Bronx. There was a, you know, I felt more comfortable, and it felt like they were actually trying… it didn’t feel like somebody was trying to quickly finish a session. It felt like they were trying to make sure that I had, um, it’s it’s kinda like how you asked the follow-up question.”
	“No matter who it was in that chain that I talked to, I never got the sense that they were just trying to get me in and get out… I always felt like they were giving me all the information that I needed for me to make a decision.”
	“So, I always trusted Navy Medicine. I heard horror stories from everybody else. And I’m like, man, that that’s, that’s not what I’m seeing… Whether’s it’s…the, um, you know, the the triage doc just gets me in the door to the actual physician or the physician assistant.”

## Data Availability

The original contributions presented in this study are included in the article. Further inquiries can be directed to the corresponding author.
